# Analysis of Application Examples of Differential Privacy in Deep Learning

**DOI:** 10.1155/2021/4244040

**Published:** 2021-10-26

**Authors:** Zhidong Shen, Ting Zhong

**Affiliations:** School of Cyber Science and Engineering, Wuhan University, Wuhan, Hubei, China

## Abstract

Artificial Intelligence has been widely applied today, and the subsequent privacy leakage problems have also been paid attention to. Attacks such as model inference attacks on deep neural networks can easily extract user information from neural networks. Therefore, it is necessary to protect privacy in deep learning. Differential privacy, as a popular topic in privacy-preserving in recent years, which provides rigorous privacy guarantee, can also be used to preserve privacy in deep learning. Although many articles have proposed different methods to combine differential privacy and deep learning, there are no comprehensive papers to analyze and compare the differences and connections between these technologies. For this purpose, this paper is proposed to compare different differential private methods in deep learning. We comparatively analyze and classify several deep learning models under differential privacy. Meanwhile, we also pay attention to the application of differential privacy in Generative Adversarial Networks (GANs), comparing and analyzing these models. Finally, we summarize the application of differential privacy in deep neural networks.

## 1. Introduction

In recent years, deep learning based on neural networks has been widely developed and successfully applied to many fields, such as image classification [1], natural language processing [2], face recognition [3, 4], interpretable mechanism learning [5], and recommendation systems [6, 7]. Deep neural networks can be trained to learn through a large number of training data. However, researches on model inference attacks [8] and model inversion attacks [9] make it easier to extract user information from the training dataset. These training samples may contain sensitive information, such as medical records, property information, biological information, and social relationships. Once leaked, it will have more or less impact on users [10]. In the era of big data, users generate numerous data every day. Once user information is collected, users often cannot control how their information is used or shared. This requires application vendors to provide policies and techniques to protect user privacy.

There are many methods to protect user information in privacy-preserving fields, such as k-anonymity [11], homomorphic encryption [12], L-diversity [13], and secure multiparty computing [14]. Most of these methods desensitize the data or encrypt it into ciphertext [15], but they are not effective for some particular attacks. For example, when performing the same query *f* = “how many boys are in the dataset” on the top 99 records and 100 records of a dataset, you can know the sex of the 100th person by comparing the two results. This is the so-called differential attack. Differential privacy is designed to defend against this attack in the first place, which provides a rigorous privacy guarantee [16]. It protects privacy by adding noise to the dataset or the results of a query function so that the query result does not increase or decrease due to the increase or decrease of a particular record [17]. The proposal of differential privacy has made a breakthrough in privacy-preserving. Differential privacy can ensure that an attacker cannot obtain private information from arbitrary records.

Differential privacy has been widely used in machine learning [18–21]. Differential privacy in deep learning is applied mostly by adding noise during the stochastic gradient descent process, as in literature [22–25]. Combining differential privacy with deep learning provides new ideas for privacy-preserving in deep neural networks [26]. Although many papers have proposed different methods to protect privacy in deep learning, there is no comprehensive paper to analyze and compare different technologies. For this purpose, this paper is proposed to compare different models of differential privacy and deep learning. After comparing and analyzing them, we classify these methods to help beginners quickly understand the knowledge in this field. We comparatively analyze and classify several deep learning models to help beginners understand the knowledge in this field.

The rest of the paper is organized as follows. This paper first introduces differential privacy and deep neural networks, as well as the types of attacks that neural networks may suffer from. We classify these models into three categories and provide a detailed introduction and comparative analysis in Section 5. At the same time, we found that differential privacy can also be applied to Generative Adversarial Networks (GAN). Two typical methods are introduced and compared in Section 6. Finally, the conclusion and discussion are made in Section 7.

## 2. Preliminaries

### 2.1. Differential Privacy

Differential privacy is a concept proposed by C. Dwork [27] in 2006 to protect statistical databases from differential attacks. For example, for a simple query *f* = “how many boys are in the database,” using this query to query in the first 99 rows of data and the first 100 rows of data, the gender of the 100th person can be inferred, which leads to the leakage of user privacy. Differential privacy can guarantee that the output results will not increase or decrease due to the increase or decrease of individual information in the database.

**Definition 1 .**

*K* is a random algorithm and *S* is a set of all possible outputs. For any two datasets, *D* and *D*′ differ from at most one different data:

(1) $$\begin{array}{c} \mathrm{Pr}\left[Κ\left[D\right]\varepsilon S\right]\leq \;\exp\left(\varepsilon \right)\times \mathrm{Pr}\left[Κ\left[D^{\prime }\right]\varepsilon S\right]. \end{array}$$

Then, algorithm *K* provides (*ε*) − differentialprivacy.

The parameter *ε* > 0, and we usually think of *ε* as 0.01, 0.1, ln 2, and ln 3.

In other words, if the algorithm works on any adjacent dataset, the probability of getting a specific output should be similar, and then we say that this algorithm can achieve the effect of differential privacy. This means that observers can hardly detect a small change in the dataset by observing the output results. This method can achieve the purpose of protecting privacy to a certain extent.

In Definition 1, *ε* is the privacy budget [28], which represents the privacy protection level provided by the algorithm *K*. The smaller *ε*, the higher the privacy protection level. There are two commonly used privacy budget composition theorems: sequential composition [29] and parallel composition [30].

**Theorem 1 (sequential composition).**

Suppose that there is a set of privacy mechanisms ℳ={ℳ_1_,…, ℳ_*m*_} performing on a dataset in sequence, and each ℳ_*i*_ provides *ε*_*i*_− differential privacy, and then the privacy mechanism ℳ will provide (*m* · *ε*_*i*_) − differential privacy.

**Theorem 2 (parallel composition).**

Suppose that there is a set of privacy mechanisms ℳ={ℳ_1_,…, ℳ_*m*_}. If each ℳ_*i*_ provides *ε*_*i*_−differential privacy on a disjointed subset of the entire dataset, then the privacy mechanism ℳ will provide (max{*ε*_*i*_,…, *ε*_*m*_}) −  differential privacy.

**Definition 2 .**

K is a random algorithm and S is the set of all possible outputs of K. For any two datasets, *D* and *D*′ differ from at most one different data:

(2) $$\begin{array}{c} \mathrm{Pr}\left[Κ\left[D\right]\varepsilon S\right]\leq \;\exp\left(\varepsilon \right)\times \mathrm{Pr}\left[Κ\left[D^{\prime }\right]\varepsilon S\right]+\delta . \end{array}$$

Then algorithm K provides (*ε*, *δ*) − differentialprivacy. When *δ*=0, algorithm *K* provides *ε*-differential privacy.

**Definition 3 .**

For a query function *f* : *D*⟶*R*^*d*^, *R*^*d*^ is a *d*-dimensional vector and adjacent datasets *D* and *D*′, and the sensitivity of *f* is defined as

(3) $$\begin{array}{c} \Delta f\;=\underset{D,D^{\prime }}{\max}{\left|\left|f\left(D\right)-f\left(D^{\prime }\right)\right|\right|}_{1},\\ =\underset{D,D^{\prime }}{\max}\sum_{i=1}^{d}\left|f{\left(D\right)}_{i}-f{\left(D^{\prime }\right)}_{i}\right|. \end{array}$$

Sensitivity is a parameter that determines how much noise is required for a particular query in the mechanism. It is only related to the type of query and considers the maximum difference between query results on adjacent datasets.

Differential privacy has different implementation mechanisms for different algorithms. The two most commonly used are the Laplace mechanism and the exponential mechanism [31]. Laplace mechanism is often used for the protection of numerical results, while the exponential mechanism is suitable for nonnumeric results.

### 2.2. Laplace Mechanism

The dense function of Laplace noise is as follows:

(4) $$\begin{array}{c} P\left(z|b\right)=\frac{1}{2b}\exp\left(-\frac{\left|z\right|}{b}\right), \end{array}$$

with variance of 2*b*^2^.

**Definition 4 .**

For the function *f* : *D*⟶*R* performing on the dataset *D*, the Laplace mechanism ℳ is defined as follows, which provides *ε*-differential privacy:

(5) $$\begin{array}{c} ℳ\left(D\right)=f\left(D\right)+\text{Lap}\left(\frac{\Delta f}{\varepsilon }\right). \end{array}$$

For a simple query *f* = “how many data in dataset satisfies the property P,” according to the above definition, the sensitivity Δ*f*  = 1. In the Laplace mechanism, let *b*=1/*ε* , then the density at *z* is proportional to *e*^−*ε*|*z*|^, and this distribution gets the maximum density at 0. For any z*z*′ with |*z* − *z*′| ≤ 1, the maximum density of *z* is *e*^*ε* ^ times than *z*′, which satisfies the definition of differential privacy [32].

### 2.3. Exponential Mechanism

Let the output domain of the query function be *R* and each value *r*∈*R* in the domain be an entity object. Under the exponential mechanism, the function *q*(*D*, *r*)⟶*R* becomes the availability function of the output value *r*, which is used to evaluate the output value the pros and cons of *r*.

**Definition 5 .**

For random algorithm ℳ with input dataset *D*, output *r*∈*R*(*M*), *q*(*D*, *r*) is the availability function in exponential mechanism, and Δ*q* is the sensitivity of the function *q* (*D*, *r*), if ℳ satisfies

(6) $$\begin{array}{c} ℳ\left(D\right)=\left(\text{return }r\propto \;\exp\left(\frac{\varepsilon q\left(D,r\right)}{2\Delta q}\right)\right). \end{array}$$

That is, if ℳ selects and outputs *r* from *R* with a probability proportional to exp(*εq*(*D*, *r*)/2Δ*q*), then ℳ gives *ε* − differential privacy.

### 2.4. Deep Neural Network

Deep neural network (DNN) is very effective for many machine learning tasks. Deep neural networks are neural networks with many hidden layers. The neural network layer inside DNN can be divided into input layer, hidden layer, and output layer. Generally, the first layer is the input layer, the last layer is the output layer, and the number of layers in the middle is all hidden layers [33]. The training process of deep neural networks is divided into forward propagation and backpropagation. Forward propagation is to use weight matrix W and the bias vector *b* to perform a series of linear operations and activation operations with the input value vector *x*. Starting from the input layer, the output of the previous layer is used to calculate the output of the next layer. The layer-by-layer backward calculation is performed until the operation reaches the output layer. The activation function makes the linear relationship between the input and the output nonlinear, which makes the DNN can approximate almost any function, making the DNN more powerful. Sigmoid function, ReLU function, tanh function, and so on are commonly used as activation function.

The loss function is defined in DNN to represent the error between the output result and the actual result, and the performance of the model can be roughly judged by the loss function. Large value of the loss function indicates that the model has insufficient fitting ability, and the coefficients need to be adjusted through backpropagation. DNN automatically adjusts the coefficients through the backpropagation algorithm. The backpropagation process compares the output result with the actual result, calculates the error between them, and propagates the error from the output layer to the front until the input layer. In the process of backpropagation, the value of the weight parameter is adjusted according to the error, so that the total loss function is reduced.

Deep neural network is divided into two phases, the training phase and the testing phase. The training phase is the above-mentioned process of forward propagation and backpropagation. After the model is trained, the model needs to be used on the test dataset to see how the model performs on the test dataset.

### 2.5. Privacy Leakage and Attacks in Deep Learning

Privacy protection in machine learning can be analyzed from confidentiality, integrity, and availability [34]. Attacks on confidentiality may expose neural network model structures, parameters, or data used to train and test models; attacks on integrity may affect the privacy of data sources; attacks on availability attempt to prevent legitimate users from accessing meaningful model output or function of the system itself. The training process of neural network requires a large amount of data, which may contain the user's personal privacy information, such as medical records, property information. Paper [35] indicates that user information can be extracted effectively from neural network. Both the training and testing phases of deep neural networks may have a privacy leakage problem. During the fine-tuning of the coefficient matrix in the training phase, the training dataset may be manipulated by the attacker. This situation is called a poison attack [36]. Poisoning attacks alter the training dataset by inserting, editing, or deleting sample points, with the goal of modifying the decision boundary of the target model.

During the testing phase of the neural network, the coefficients of the model have been determined, and attackers can also attack the model. There are three main types of attacks, model extraction attacks, model inversion attacks, and member inference attacks. Model extraction attacks are to infer the specific parameters or structure of the model by the attacker through the model test results. Assuming that the model has *n* parameters, the attacker can test the model with *m* (*m* > *n*) samples, list the test results and input samples into *m* linear equations, and solve the equations to get *n* parameter values [35].

Model inversion attacks mean that the attacker can extract information related to the training data from the model test results, such as the sensitive characteristics of the training data [9]. In face recognition, an attacker can randomly construct a picture, target a certain sample (such as tom) in the training dataset, and use the gradient descent method to randomly modify the prediction result to obtain a picture with tom's face features.

Member inference attacks aim to infer whether a particular record is in the training dataset [8]. There are black-box attacks and white-box attacks [22].

  Black-box attacks: the attacker can only get the output of the model with arbitrary inputs. For any input *x*, the attacker can only obtain *f* (*x*; *W*) but cannot get the parameter matric *W* of the model and the intermediate steps of the calculation. An attack against a black-box model has been defined in [8] which exploits the statistical difference between the model's predictions on the training set and unseen data.
  White-box attacks: for any input *x*, besides the output, the attacker can also obtain the structure and parameters of the model and observe the intermediate calculation steps in the hidden layer.

### 2.6. Differential Privacy in Deep Learning

Due to the privacy leakage in the neural network, it is necessary to protect these models. The proposal of differential privacy makes a new way to protect privacy [37]. In this section, we analyze six differential privacy models: DP-SGD, the improved DP-SGD, Adaptive Laplace Mechanism, dPA, PCDBN, and PATE. We divide these models into three categories. The first category is to increase noise during the stochastic gradient descent of neural networks to achieve differential privacy. DP-SGD improved DP-SGD, and the Adaptive Laplace Mechanism belongs to the first category. The second category is based on functional mechanisms, which achieve differential privacy by perturbing the objective function of the optimization problem instead of its result. dPA and PCDBN belong to the second category. The third category is a new framework that protects privacy through knowledge aggregation and transmission [38]. PATE model belongs to the third category.

### 2.7. The First Category

#### 2.7.1. DP-SGD

DP-SGD (Differential privacy-Stochastic Gradient Descent) algorithm was proposed by Martín Abadi et al. in 2016 [23]; this algorithm aims to control the impact of the training dataset on the training process, especially during the calculation of gradient. Based on [24], compared to the algorithms in this paper, the DP-SGD algorithm has been modified and expanded, especially in the calculation of privacy budget. The main idea of DP-SGD is shown in Algorithm 1.

For the Gaussian distribution noise in this algorithm, when $\sigma =\sqrt{2\;\log1.25/\delta }/\varepsilon$, then every single step suffices (*ε*, *δ*) − differential privacy [31]. Since each group of the algorithm is composed of a randomly selected sample, privacy amplification theorem [39] indicates that it gives (*O*(*qε*), *qδ*) − differential privacy, *q*=*L*/*N* is the probability of randomly selecting samples, and *ε* ≤ 1.

For the DP-SGD algorithm, an important issue is to track the privacy budget in training phases. This paper proposes a privacy accounting method “Moments Accountant,” which can prove that this algorithm satisfies (*O*(*qε*√*T*), *qδ*)− differential privacy. The bound of privacy cost using Moments Accountant mechanism is less than the bound using strong composition theorem [40]. We save a factor of $\sqrt{\text{log}\left(1/\delta \right)}$ in *ε* part and a factor of *Tq* in *δ* part.

#### 2.7.2. Improved DP-SGD

There are two main problems with the DP-SGD algorithm. The first problem is that when implementing the algorithm, in order to obtain higher efficiency, random shuffling is often used to batch data. This method will lead to higher privacy loss and make the privacy loss calculated by Moment Account underestimated. The second problem is that the DP-SGD algorithm needs to iterate multiple times when calculating the privacy loss. To solve these problems, Lei Yu et al. [42] proposed a method for training neural networks by using concentrated differential privacy (CDP) [43]. CDP makes the privacy protection algorithm more practical than traditional DP when doing a lot of calculations, while still providing a strong privacy guarantee. This paper first proposes a dynamic privacy budget allocation technique and then develops zCDP-based privacy accounting methods for different data batch processing methods.

### 2.8. Dynamic Privacy Budget Allocation

For a given privacy budget, the accuracy of the final model depends on how the privacy budget is allocated during training. Privacy budget allocation technology aims to optimize the budget allocation in the training process, so as to obtain a differential privacy DNN model with higher accuracy. The main idea is that as the model accuracy converges, the noise on the gradient becomes less. This will make the model closer to the optimal solution while having higher accuracy. On this basis, literature [42] proposed two privacy budget allocation techniques: adaptive schedule based on public validation dataset and predefined schedules.

### 2.9. Adaptive Schedule Based on Public Validation Dataset

The technique uses the public verification dataset to monitor verification errors during training and reduce the noise scale when verification errors stop improving. Specifically, whenever the verification accuracy is improved to less than the threshold *δ*, the noise level is reduced by a factor of *k* until the privacy budget is exhausted.

### 2.10. Predefined Schedules

This method does not use a verification dataset but predefines certain decay functions so that the noise level will decrease over time. This document mainly uses four decay functions to reduce the noise level: Time-Based Decay, Exponential Decay, Step Decay, and Polynomial Decay.

### 2.11. Privacy Accountant

**Definition 6 .**

CDP considers privacy loss on an outcome *o* of the randomized mechanism A operating on two adjacent databases *D* and *D*′ as follows:

(7) $$\begin{array}{c} L_{\left(\left.Α\left(D\right)\right\|A\left(D^{\prime }\right)\right)}^{\left(o\right)}=\log\frac{\mathrm{Pr}\left(Α\left(D\right)=o\right)}{\mathrm{Pr}\left(Α\left(D^{\prime }\right)=o\right)}, \end{array}$$

(*µ*, *τ*)-CDP ensures that the mean of privacy loss does not exceed *µ*, and the probability of the loss exceeding its mean by an amount of *t* · *τ* is bounded by *e*^−*t*^2^/2^ [43]. Bun and Steinke [44] proposed another form of (*µ*, *τ*)-CDP, called zero-concentrated differential privacy, zCDP.

**Definition 7 .**

For two datasets differs from only one data, random mechanism *A* is *ρ* − zCDP if

(8) $$\begin{array}{c} D_{\alpha }\left(\left.Α\left(D\right)\right\|A\left(D^{\prime }\right)\right)=\frac{1}{\alpha -1}\log\left(E\left[e^{\left(\alpha -1\right)L^{\left(o\right)}}\right]\right)\leq \rho \alpha , \end{array}$$

*αε*(1, *∞*), *D*_*α*_(Α(*D*)‖*A*(*D*′)) is *α*-*Re*′nyi divergence between *D* and *D*′.

There are two propositions proposed in literature [44].

**Proposition 1 .**

For any *δ* > 0, if A satisfies *ρ* − zCDP, then A satisfies $\left(\rho +2\sqrt{\rho \;\log\left(1/\delta \right)},\delta \right)$-DP.

**Proposition 2 .**

The mechanism with *N*(0, Δ^2^*σ*^2^*I*) Gaussian noise satisfies (1/2*σ*^2^) − zCDP.

Since zCDP and DP are comparable, this paper proposes a privacy accounting method based on zCDP. According to the sequential composition satisfied by zCDP, if each iteration satisfies *ρ* − zCDP and the total number of iterations of the training process is *T*, then the entire training process satisfies (*Tρ*) − zCDP. This paper proposes the composition of privacy loss under two common batch processing methods: replacement random sampling and random shuffling. Replacing random sampling refers to randomly sampling each sample from the training dataset. Random shuffling means that the training dataset is randomly shuffled into batches of similar size, and the SGD process processes one batch at a time. Random shuffle is a common practice, its performance is better than random sampling, and the convergence speed is improved [45].

In the random shuffling method, the loss of privacy is tracked using Theorem 2. In replacing random sampling method, Theorem 4 is used to calculate.

**Theorem 4 .**

Let $\hat{\rho }=P\left(q,\sigma \right)$ and *u*_*α*_=*U*_*α*_(*q*, *σ*). The mechanism Α′ has

(9) $$\begin{array}{c} D_{\alpha }\left(\left.{Α}^{\prime }\left(D\right)\right\|{Α}^{\prime }\left(D'\right)\right)\leq \alpha \hat{\rho }. \end{array}$$

For 1 < *α* ≤ *u*_*α*_, it satisfies

(10) $$\begin{array}{c} \left\{\begin{array}{cc} \left(\hat{\rho }+2\sqrt{\hat{\rho }\log\left(1/\delta \right)},\delta \right)-DP\;, & \text{if }\delta \geq 1/\exp\left(\hat{\rho }{\left(u_{\alpha }-1\right)}^{2}\right),\\ \left(\hat{\rho }u_{\alpha }-\frac{\log\;\delta }{u_{\alpha }-1},\delta \right)-DP\;, & \text{otherwise,} \end{array}\right. \end{array}$$

where *q* is the probability*L*/*N* of randomly selected samples and *P*(*∗*) and *U*(*∗*) are functions of *q* and *δ*.

Under different privacy budget allocation methods, given the data processing method, the privacy loss can be calculated according to Theorem 2 or Theorem 3.

#### 2.11.1. Adaptive Laplace Mechanism

Paper [46] proposed an Adaptive Laplace Mechanism (AdLM) whose main idea is to add more noise to features that are not related to the model output. According to the contribution of each feature to the model output, the Laplace noise is injected into features adaptively. Unlike the method in [47], the noise and privacy budget injected by this method are not accumulated in each training step. The consumption of the privacy budget is independent of the number of training times. The Adaptive Laplace Mechanism is shown in Algorithm 2.

The computing loss function *F*_*L*_(*θ*_*t*_) suffices *ε*_3_ − differential privacy, according to composition theorem [48]; this algorithm gives (*ε*_1_+*ε*_2_+*ε*_3_)-differential privacy. Besides, this mechanism can be used in various deep learning models, such as CNN [49], deep autoencoder [33], and convolution deep belief networks [50].

#### 2.11.2. Comparative Analysis

Among the three models of the first type, DP-SGD uses gradient clipping and increased Gaussian noise to implement differential privacy. At the same time, a privacy accounting mechanism “Moments Accountant” is proposed. The privacy loss threshold obtained using this mechanism is small. On this basis, Lei Yu et al. [42] improved the DP-SGD algorithm and proposed a dynamic privacy budget allocation technique. Through experiments in [42], the comparison of the privacy accounting method of DP-SGD and the improved DP-SGD algorithm is shown in Table 1. It can be seen that the privacy loss obtained by the MA method and the zCDP method is smaller than that using the strong composition, indicating that both methods can obtain the privacy loss value more accurately. zCDP(RF) has a higher privacy loss value than MA and zCDP(RS) methods because RS introduces more certainty. However, RF is a more commonly used method in deep neural networks. The DP-SGD algorithm is also implemented using RF for batch processing. It can be seen that the MA method underestimates its privacy loss.

As the accuracy of the model converged, the noise on the gradient became less. At the same time, zCDP-based privacy accounting methods are developed for different batch processing methods. The Adaptive Laplace Mechanism adds more noise to features that are not related to the model output. According to the contribution of each feature to the model output, the Laplace noise is adaptively injected into the feature. The accuracy comparison of these three models is shown in Table 2.

It can be seen that, on the MNIST dataset, the accuracy of the three models is very high, reaching more than 90%. When *ε*=0.5, the accuracy of the AdLM model is slightly higher than that of DP-SGD, reaching 93.66%. On the CIFAR-10 dataset, the improved algorithm of DP-SGD has lower accuracy, and the accuracy of DP-SGD and AdLM algorithms is higher. When *ε*=8, the accuracy of AdLM algorithm is more accurate than that of the DP-SGD algorithm. The rate is about 4% higher. The accuracy of the improved DP-SGD method under different privacy budget decay functions is slightly different, but similar. From the perspective of convergence speed, the DP-SGD algorithm is faster than AdLM. The improved algorithm of DP-SGD is proposed for the problem of excessive iterations and underestimation of privacy loss when calculating the privacy loss of the DP-SGD algorithm. Therefore, its convergence speed is faster than DP-SGD.

### 2.12. The Second Category

#### 2.12.1. dPA

Deep autoencoder (dA) is one of the basic deep learning models and is widely used in natural language processing and other fields [33]. Autoencoder is an unsupervised learning algorithm, which is mainly used for data dimensionality reduction or feature extraction. In deep learning, it can be used to determine the initial value of the weight matrix before training starts. It encodes the high-dimensional input so that the compressed low-dimensional vector maintains the characteristics of the input data. Phan et al. [51] proposed a deep private autoencoder (dPA), which is implemented by perturbing the target function (not the result) of a traditional deep autoencoder to achieve differential privacy. This algorithm is improved on the basis of functional mechanism (FM) [20]. The algorithm steps are as follows.

Derive polynomial approximation of data reconstruction function RE(*D*, *W*), denoted as $\hat{\text{RE}}\left(D,W\right)$.

For a given encoding $\tilde{x_{i}}$, the reconstruction function of the autoencoder is as follows:

(11) $$\begin{array}{c} \text{RE}\left(t_{i},\text{W}\right)=-\log\;P\left(x_{i}|\tilde{x_{i}},W\right),\\ =-\sum_{j=1}^{d}\left(x_{ij}\log\tilde{x_{ij}}\right)+\left(1-x_{ij}\right)\text{log}\left(1-\tilde{x_{ij}}\right). \end{array}$$

The above function is transformed into Taylor expansion:

(12) $$\begin{array}{c} \hat{RE}\left(D,W\right)=\sum_{i=1}^{\left|D\right|}\sum_{j=1}^{d}\left(\sum_{l=1}^{2}f_{ij}^{\left(0\right)}\left(0\right)+\left(\sum_{l=1}^{2}f_{ij}^{\left(1\right)}\left(0\right)\right)W_{j}h_{i}+\left(\sum_{l=1}^{2}\frac{f_{ij}^{\left(2\right)}\left(0\right)}{2!}\right){\left(W_{j}h_{i}\right)}^{2}\right). \end{array}$$

The function $\hat{\text{RE}}\left(D,W\right)$ is perturbed by using functional mechanism, denoted as $\bar{\text{RE}}\left(D,W\right)$.

Calculate the sensitivity of $\hat{RE}\left(D,W\right)$ and $\hat{RE}\left(D^{\prime },W\right)$, and perturb the function according to the sensitivity. The sensitivity is as follows:

(13) $$\begin{array}{c} \Delta =2\underset{t}{\max}\sum_{j=1}^{d}\sum_{R=0}^{2}\left\|\lambda_{jt}^{\left(R\right)}\right\|\leq d\left(b+\frac{1}{4}b^{2}\right). \end{array}$$

Compute $\bar{W}=\arg\min_{w}\;\bar{\text{RE}}\left(D,W\right)$ to get the initial weight matrix of the input layer.

### 2.13. Private Autoencoder (PA) Stacking

Fix the initial weight matrix of the input layer to autoencoder each subsequent layer. The hidden units of the lower layer will be considered as the input of the next PA. To guarantee that this input to the next PA satisfies $\sqrt{\sum_{j=1}^{b}h_{ij}^{2}}\leq 1$, a normalization layer is added on the top of the hidden layer to make ${𝔥}_{ij}=h_{ij}-\gamma_{j}/\left(\varphi_{j}-\gamma_{j}\right)\cdot \sqrt{b}$.

Derive and perturb the polynomial approximation of cross-entropy error *𝒞*(*θ*), denoted as $\bar{𝒞}\left(\theta \right)$.

The cross-entropy error function of the softmax layer is

(14) $$\begin{array}{c} C\left(Y_{T},\theta \right)=-\sum_{i=1}^{\left|Y_{T}\right|}\left(y_{i}\log\left(1+e^{-W_{\left(k\right)}h_{i\left(k\right)}}\right)+\left(1-y_{i}\right)\log\left(1+e^{W_{\left(k\right)}h_{i\left(k\right)}}\right)\right). \end{array}$$

The above function is transformed into the polynomial form:

(15) $$\begin{array}{c} \hat{C}\left(Y_{T},\theta \right)=\sum_{i=1}^{\left|Y_{T}\right|}\sum_{l=1}^{2}\sum_{R=0}^{2}\frac{f_{l}^{\left(R\right)}\left(0\right)}{R!}{\left(W_{\left(k\right)}h_{i\left(k\right)}\right)}^{R}. \end{array}$$

Calculate the sensitivity Δ_*C*_=|*𝔥*_(*k*)_|+1/4|*𝔥*_(*k*)_|^2^ of $\hat{C}\left(Y_{T},\theta \right)$ and $\hat{C}\left(Y_{T}^{\prime },\theta \right)$, and perturb the cross-entropy loss function according to the sensitivity.

Computer $\bar{\theta }=\arg\min_{\theta }\bar{𝒞}\left(\theta \right)$; return $\bar{\theta }$.

#### 2.13.1. pCDBN

Private deep convolutional belief network (pCDBN) [52] is essentially a differential privacy version of convolutional deep belief network (CDBN) [50]. This method is similar to dPA in [51], but there are still some differences. Since the global sensitivity of the CDBN in the functional mechanism cannot be derived, it is difficult to identify the approximate error range in the CDBN, and the Chebyshev polynomial is used in the pCDBN to approximate the nonlinear objective function. Then, noise is injected into these polynomials, and the functional mechanism is used to make each hidden layer's training phase satisfy *ε*-differential privacy. Finally, the hidden layer becomes a private hidden layer after the above transformation, the private hidden layer is stacked on each layer, and the polynomial form of the cross-entropy error function of the softmax layer is obtained and then perturbed to generate a private convolutional deep confidence network. The algorithm steps are as follows.

Derive a polynomial approximation of the energy function $E\left(D,W\right),\text{ denoted as }\hat{E}\left(D,W\right)$.

Perturb function $\hat{E}\left(D,W\right)$ by using functional mechanism [20], denoted as $\bar{E}\left(D,W\right)$. Stack the private hidden and pooling layers (*H*, *P*) to construct pCDBN. Apply the technique presented in [51], and the cross-entropy error is transformed into a polynomial form at the softmax layer of the classification and prediction tasks and then perturbed.

## 3. Experiment

**Figure 1:**
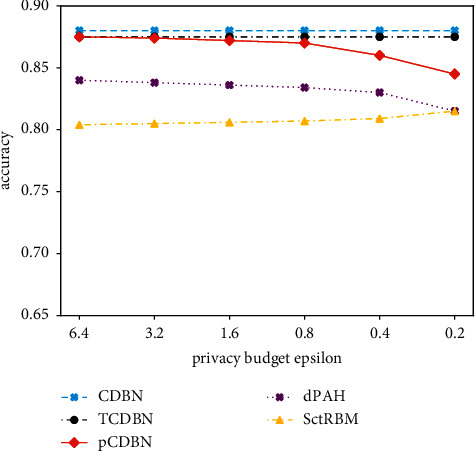
Comparison of different models [40].

Experimental comparison between pCDBN, CDBN, dPAH (dPA for human behavior prediction), TCDNB (a simplified version of CDBN, without adding noise to the energy function approximation), and conditionally restricted Boltzmann machine [53] (SctRBM) is shown in Figure 1. It can be seen that the accuracy of the model without privacy-preserving remains basically unchanged. Due to the addition of noise, dPAH and pCDBN have lower accuracy than CDBN and TCDNB models. The accuracy of pCDBN is higher than that of the dPAH model, and the accuracy of the pCDBN model is even higher than that of SctRBM without added noise.

**Figure 2:**
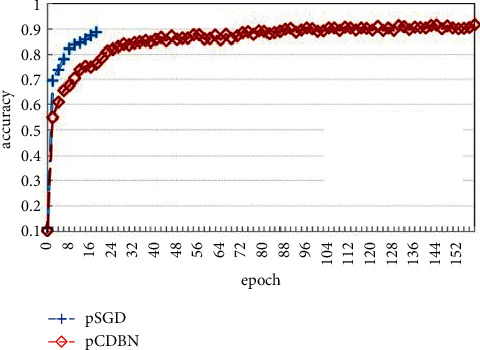
Comparison of DP-SGD and pCDBN [40].

This literature also compares the pCDBN model with the DP_SGD (pSGD) model in [23]. The experimental results are shown in Figure 2. When *ε*=0.5, the pSGD model reached 88.75% accuracy at 18 epochs, and the pCDBN model pCDBN reached 91.71% accuracy after 162 epochs, which was higher than the pCDBN model accuracy.

### 3.1. Comparative Analysis

In the second type of model, based on the functional mechanism, dPA uses Taylor expansion to approximate the cross-entropy error function to a polynomial form and then injects noise. pCDBN uses Chebyshev polynomials to derive polynomial approximations of the energy function and nonlinear objective functions and then injects noise. The algorithm and ideas of these two models are basically similar, but they use different methods to transform the objective function into a polynomial form. Literature [52] compared pCDBN with dPAH, a human behavior recognition model using dPA, in the experimental part. The results can be seen in Figure 2. The result shows that the accuracy of pCDBN is higher than that of dPAH.

## 4. The Third Category

### 4.1. PATE

#### 4.1.1. Framework

**Figure 3:**
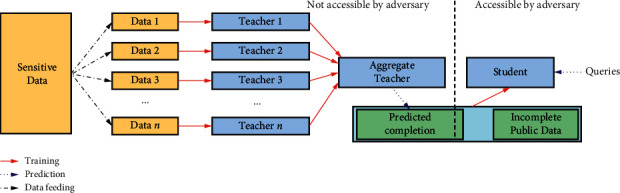
Framework of PATE [54].

Some neural network models may inadvertently remember some privacy data, and there is a risk of leaking privacy. The PATE (Private Aggregation of Teacher Ensembles) algorithm proposed by Papernot et al. [54] can provide a strong guarantee for training data. This method combines multiple models trained using disjoint datasets (such as records from different subsets of users) in a black-box manner. These models are not published but are used as “teachers” for “student” models. Students will learn to predict the output selected by noise voting among all teachers and have no direct access to individual teachers, basic data, or parameters. The general framework of the PATE algorithm is shown in Figure 3, which is reproduced from Nicolas Papernot et al., 2017 [54].

This algorithm divides the training dataset into *n* groups, and each group is trained using *n* models, and these *n* models become “teacher” models. When the prediction results of these *n* teacher models are combined, it is performed according to the principle that the minority obeys the majority, and noise is added to it, thereby disturbing the voting result and protecting privacy. For an input *x*, if most teachers' predictions are consistent, adding noise will not affect the final prediction result; if the teacher's prediction results are divided into two categories and the number of votes is the same, then one of the predictions will be output randomly after adding the noise.

As the number of predictions increases, the model needs to add more noise, which makes the model useless. And if the adversary can access the parameters of the model, the privacy guarantee cannot be held. To solve this problem, the PATE algorithm introduces a “student” model. The student model is trained using nonsensitive data and unlabeled data. Part of the unlabeled data is labeled by the teacher model and then used as the training dataset for the student model with the remaining unlabeled data. Using the student model instead of the aggregation of teacher for deployment, a fixed loss of privacy can be obtained, the value of which is determined by the number of queries made to the teacher model during student model training. Therefore, even if the adversary obtains its architecture and parameters by attacking the student model, the algorithm can protect user privacy from being leaked.

The PATE framework uses the Moment Account mechanism of [23] to conduct privacy analysis. At each step, add Lap(1/*γ*) to the aggregation mechanism to implement(2*γ*, 0) − *DP*, and then after *T* step, this mechanism is implemented $\left(4T\gamma^{2}+2\gamma \sqrt{2T\;\ln1/\delta },\delta \right)$-DP. The accuracy of the student models on the MNIST and SVHN datasets is shown in Table 3. It can be seen that the PATE model achieves up to 98% accuracy in the MNIST dataset and more than 90% accuracy in the SVHN dataset.

### 4.2. Experiment

In the third type of model, PATE uses the aggregation results of the “teacher” model to train the “student” model, so that attackers cannot directly access the “teacher” model, private data, or model parameters. PATE supports various models flexibly, especially for deep neural networks. Experiments show that the PATE model has higher accuracy on the MNIST and SVHN datasets, and 98% accuracy on the MNIST dataset (*ε*=2.04, *δ*=10^−5^) as shown in Table 3.

## 5. Comparative Analysis of These Three Categories

In the three types of methods, noise is added to achieve differential privacy. The first category of methods adds noise to the model gradient. On this basis, it discusses how to assign privacy (dynamic or static) and how to add noise (fixed or not). The second category is based on functional mechanisms and protects privacy by perturbing the objective function of the optimization problem rather than its result. The third type is a new framework by training the teacher model dispersedly, making decisions based on the prediction results of the teacher model and the noise added to it, and then introducing the student model to hold the privacy guarantee. These three types of methods can realize the use of differential privacy in DNN to protect user privacy data, which is representative. The comparative analysis of these three types of models is shown in Table 4.

### 5.1. Differential Privacy IN GAN

Generative Adversarial Network (GAN) is a model used to estimate the distribution of the training dataset and use this distribution to randomly generate samples [55]. However, due to the high complexity of the model, it can easily remember the training samples, which leads to the leakage of user privacy data. By repeatedly sampling from the distribution, there is a considerable opportunity to recover the training samples. For example, Hitaj et al. [56] introduced an active inference attack model that can reconstruct training samples from the generated samples. The general idea of protecting user privacy information using differential privacy in a GAN is to add noise to the discriminator during the training process and cooperate with the calculation of the generator, such as the literature ([57–59]). Reference [59] proposed an AC-GAN model for clinical data sharing, and the model does not leak user privacy data. Now we will introduce some methods of using differential privacy to protect GAN.

### 5.2. DPGAN

#### 5.2.1. Algorithm

Liyang et al. [57] proposed a framework that combines the differential privacy method in [23] with GAN and DPGAN. This model adds carefully designed noise during the training process, performs gradient clipping, and uses the Wasserstein distance [60] as an approximation of the distance between probability distributions, which is more reasonable than the JS-divergence in GAN. Specifically, when calculating the *D* gradient relative to the actual sample *x*, we first clip the gradient by injecting the designed noise (line 6) to ensure that the sensitivity is limited by *e*. Then, we add random noise sampled from the Gaussian distribution. RMSProp is an optimization algorithm that can adaptively adjust the learning rate according to the size of the gradient [61]. The detailed algorithm is shown in Algorithm 3.

The clip function in Algorithm 3 satisfies that the activation function of the discriminator has a bounded range and bounded derivatives everywhere:*σ*(·) ≤ *B*_*σ*_ and *σ*′(·) ≤ *B*_*σ*′_, and every data point *x* satisfies ‖*x*‖ ≤ *B*_*x*_ and then ||*g*_*w*_(*x*^(*i*)^, *z*^(*i*)^)|| ≤ *c*_*g*_ for some constant *c*_*g*_.

When *q*=*m*/*M*, noise scale $\sigma_{n}=2q\sqrt{n_{d}\text{log}\left(1/\delta \right)}/\varepsilon$, and Algorithm 3 is (*ε*, *δ*) − *DP*. DPGAN's privacy loss is independent of the amount of data generated, which makes this method suitable for various real-world situations.

## 6. Experiment

**Figure 4:**
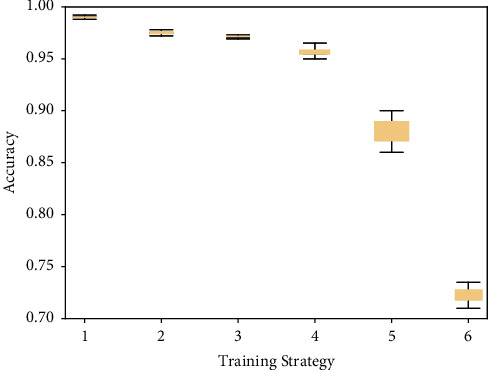
Accuracy of DPGAN on digits 4 and 5 [46].

The accuracy of DPGAN under the MNIST dataset is shown in Figure 4. From left to right on the figure are the real data, the generated nonprivate samples, and the generated samples where *ε* = 11.5, 3.2, 0.96, and 0.72, respectively. As can be seen from this figure, as the noise level increases, the accuracy of the generated samples is also higher, indicating that the more efficient the samples are generated.

### 6.1. GANobfuscator

#### 6.1.1. Framework

**Figure 5:**
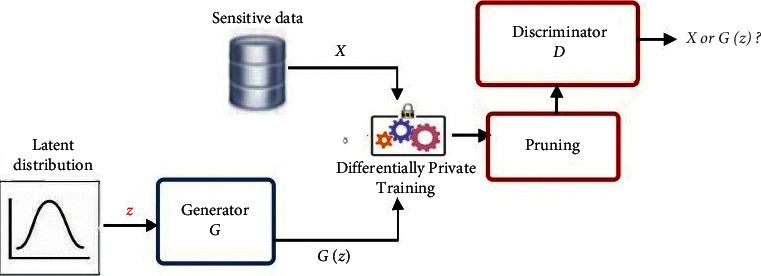
Framework of GANobfuscator [47].

GANobfuscator is a model proposed by Chugui Xu et al. [58] which uses differential privacy to mitigate the leakage of private information in GANs. This algorithm adds well-designed noise to the learning process of GANs. With this algorithm, analysts can generate unlimited synthetic samples for any task without leaking information about the training samples. The general framework of this algorithm is in Figure 5.

The privacy data *X* reaches the discriminator *D* through the privacy protection layer. The role of the discriminator is to distinguish the real data from the artificial dataset $\tilde{X}$ generated by the training differential privacy generator G. The implementation method of GANobfuscator is similar to the method in [23], which adds noise during the training process. Compared with the discriminator *D* and the generator *G*, *G* generally uses the construction module [62] and batch normalization [63] to generate samples. *D* has only a simple structure and a small number of parameters and *D* can get real data directly. Therefore, *D* is easier to measure the loss of privacy. Noise only needs to be added when training the discriminator *D*.

## 7. Algorithm

The algorithm flow of GANobfuscator is similar to the DPGAN algorithm. The difference between the two lies in the clip function when clipping gradient (see Algorithm 3). The problem brought by Algorithm 3 is that the quality of the generated samples is low and the model convergence speed is slow. In order to solve this problem, Chugui Xu et al. [58] designed an optimized GANobfuscator algorithm. This method enhances GANobfuscator through adaptive pruning function to monitor the change of gradient and dynamically adjust the pruning range to converge faster and get stronger privacy. The optimized GANobfuscator algorithm is shown in Algorithm 4.

### 7.1. Experiment

**Figure 6:**
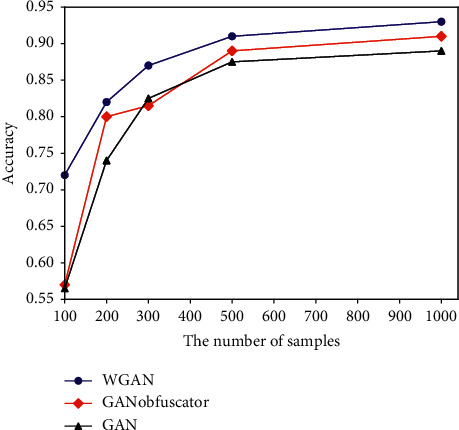
Accuracy of GANobfuscator [47].

When *ε*=2, *δ*=10^−5^, randomly select different numbers of samples in the generated data, establish a classifier, and then use the MNIST dataset for testing. After repeating 100 times, the experimental accuracy is obtained as shown in Figure 6. Experimental results show that the accuracy of the GANobfuscator model is higher than that of the GAN without noise, and this model greatly increases the number of samples, making the availability of the generated model high.

### 7.2. Comparative Analysis

**Figure 7:**
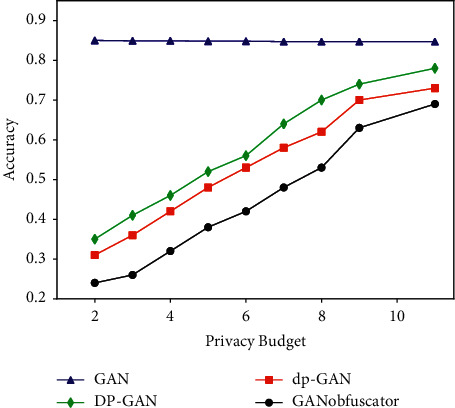
Precision of the inference attack [47].

Both the GANobfuscator and the DPGAN model are based on the WGAN model and add noise to the gradient in the discriminator training process to achieve differential privacy. Their difference lies in the way in which the noise is clipped. DPGAN pruning guarantees that {*f*_*w*_(*x*)}_*w*∈*W*_ are all *K*_*w*_ − Lipschitz and limits the gradient of each data point in a way. GANobfuscator monitors the change in a gradient through adaptive pruning and dynamically adjusts the pruning range to achieve faster convergence and stronger privacy. The author of [58] conducted an experiment on the ability of the model to resist inference attacks. The experimental results are shown in Figure 7. It can be seen that, under the CelebA dataset, the GANobfuscator model has a stronger ability to resist inference attacks than the GAN, dp-GAN, and DP-GAN models.

## 8. Conclusion

In the current era of information explosion, the widespread application of deep learning makes user privacy easy to leak. The development of differential privacy technology provides new ideas for privacy protection in deep neural networks (DNNs). Using differential privacy to protect data in DNNs is usually achieved by adding noise during the stochastic gradient descent process. We compared and analyzed several examples of combining differential privacy with DNNs, and then we classified them. The application of differential privacy in deep learning is classified into three categories. The first category adds noise to the model gradient. On this basis, it discusses how to assign the privacy budget and how to add noise. The second type is based on the functional mechanism, adding noise to the objective function instead of its result. And the third is a new framework designed to support various models flexibly. It relies on the aggregation and noise of multiple teacher models to make decisions. We also pay attention to the application of differential privacy in Generative Adversarial Network: GANobfuscator and DPGAN. They are implemented by adding noise to the discriminator, but their gradient clipping methods are different.

Although the application of differential privacy in deep learning is currently in its infancy, it is potential and many methods are worth exploring. Differential privacy can be widely applied in various scenarios that require privacy protection, such as recommendation systems, face recognition, and action recognition. In the future, differential privacy may be combined with federated learning and transfer learning or defend against adversarial attacks to improve the robustness of the model.

## Figures and Tables

**Algorithm 1:**
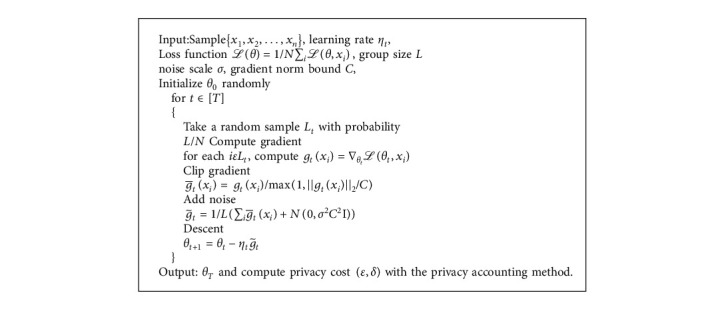
DP-SGD.

**Algorithm 2:**
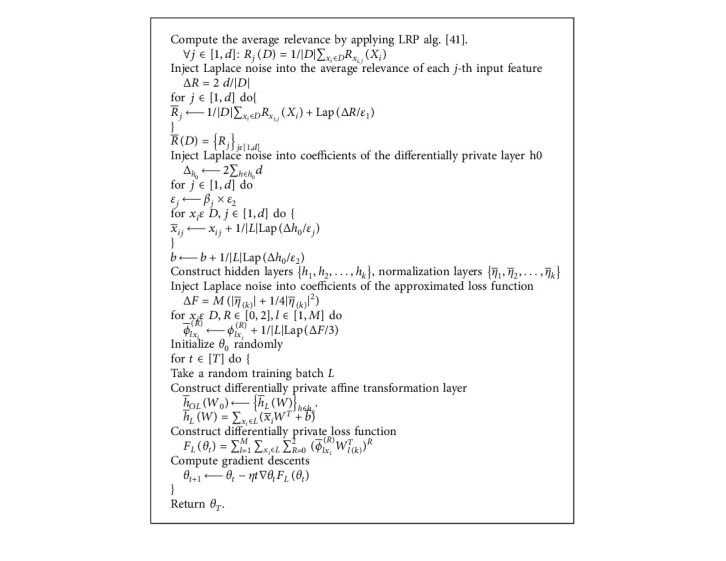
Adaptive Laplacian mechanism.

**Algorithm 3:**
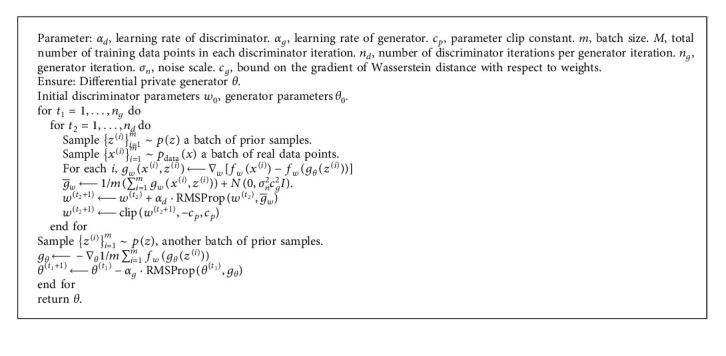
DPGAN.

**Algorithm 4:**
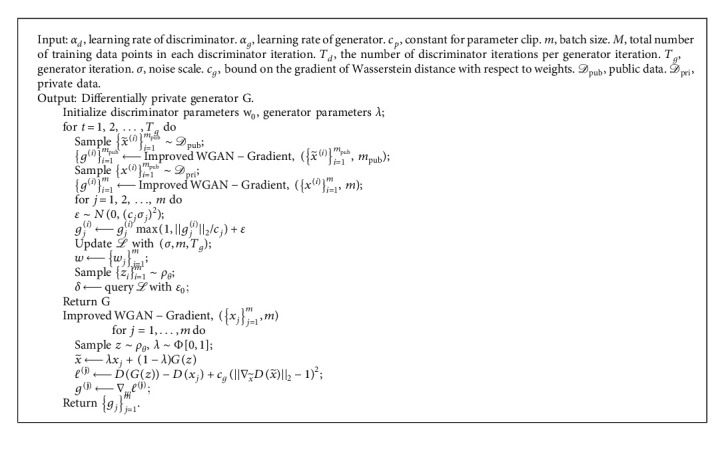
Optimizing GANobfuscator.

**Table 1:** Comparison of privacy accounting methods.

Mechanism	Privacy accounting approaches	*q*	*σ*	*δ*	Epoch	*ε*
Baseline	Strong composition	0.01	6	10^−5^	400	34.3
DP-SGD	Moment Account (MA)					1.67
The improvement of DP-SGD	zCDP (random sampling, RS)					2.37
	zCDP (random reshuffling, RF)					21.5

**Table 2:** Comparing the accuracy of the first category.

Mechanism	Dataset		Parameters	Epoch	Accuracy (%)
DP-SGD	MNIST		*ε*=0.5, *δ*=10^−5^	16	90
			*ε*=2, *δ*=10^−5^	140	95
			*ε*=8, *δ*=10^−5^	800	97
	CIFAR-10		*ε*=2; *δ*=10^−5^	60	67
			*ε*=4; *δ*=10^−5^	130	70
			*ε*=8; *δ*=10^−5^	700	73

The improvement of DP-SGD	MNIST	Time	*k* = 0.05	38	93.4
		Step	*k* = 0.6; period = 10	31	92.8
		Exp	*k* *=* 0.01	71	93.4
		Poly	*k* *=* 3; *σ*_end_=2; period = 100	44	93.0
		Validation	*k* = 0.7; *m* = 5; *σ*=0.01; period = 10	64	93.0
	CIFAR-10	Exp	*σ*_0_=30; *k* = 0.001	200	43
		Validation	*σ*_0_=35; *k* = 0.99; period = 50; *m* = 1; *δ*=0.01	200	45

AdLM	MNIST		*ε*=0.25	500	90.2
			*ε*=0.5	500	93.66
	CIFAR-10		*ε*=2.5	800	72.1
			*ε*=8	800	77

**Table 3:** Accuracy of PATE reproduced from Nicolas Papernot et al. [54].

Dataset	*ε*	*δ*	Queries	Nonprivate baseline (%)	Student accuracy (%)
MNIST	2.04	10^−5^	100	99.18	98.00
MNIST	8.03	10^−5^	1000	99.18	98.10
SVHN	5.04	10^−5^	500	92.80	82.72
SVHN	8.19	10^−5^	1000	92.80	90.66

**Table 4:** Comparison of DP-models.

Model	Similarity	Difference
DP-SGD	Add noise to gradient, they are DP-SGD algorithm, and it is variant	Add Gaussian noise to SGD Moments Accountant
The improvement of DP-SGD		Dynamic privacy budget allocation privacy accounting methods for different batch processing methods
Adaptive Laplace Mechanism		Adaptively add more noise to features that are not related to the model output
dPA	Based on functional mechanism, approximate functions to polynomial forms and perturb objective function mainly used for optimal algorithm	Uses Taylor expansion to approximate the cross-entropy error function to a polynomial form and add noise
PCDBN		Use Chebyshev polynomials to derive polynomial approximations of nonlinear objective functions and add noise
PATE		Decentralized training model adds noise to decisions and introduces student models
